# A foot and mouth disease ranking of risk using cattle transportation

**DOI:** 10.1371/journal.pone.0284180

**Published:** 2023-04-13

**Authors:** Fausto Moreno, Juan Galvis, Francisco Gómez

**Affiliations:** 1 Facultad de Medicina Veterinaria y de Zootecnia, Departamento de Producción Animal, Universidad Nacional de Colombia, Bogotá, Colombia; 2 Facultad de Ciencias, Departamento de Matemáticas, Universidad Nacional de Colombia, Bogotá, Colombia; 3 Laboratorio de Analítica de Datos (Datalab), Universidad Nacional de Colombia, Bogotá, Colombia; Texas A&M University College Station, UNITED STATES

## Abstract

Foot-and-mouth disease (FMD) is a highly infectious condition that affects domestic and wild cloven-hoofed animals. This disease has substantial economic consequences. Livestock movement is one of the primary causes of disease dissemination. The centrality properties of the livestock mobilization transportation network provide valuable information for surveillance and control of FMD. However, the same transportation network can be described by different centrality descriptions, making it challenging to prioritize the most vulnerable nodes in the transportation network. This work considers the construction of a single network risk ranking, which helps prioritize disease control measurements. Results show that the proposed ranking constructed on 2016 livestock mobilization data may predict an actual outbreak reported in the Cesar (Colombia) region in 2018, with a performance measured by the area under the receiver operating characteristic curve of 0.91. This result constitutes the first quantitative evidence of the predictive capacity of livestock transportation to target FMD outbreaks. This approach may help decision-makers devise strategies to control and prevent FMD.

## Introduction

Foot-and-mouth disease (FMD) is a highly infectious condition that affects domestic and wild cloven-hoofed animal species, including cattle, sheep, goats, and pigs [1]. This disease has devastating economic consequences, resulting from the restrictions on international trade of animals and animal products from infected countries, as well as the high costs associated with the implementation of outbreak controlling mechanisms [2, 3].

FMD transmission factors include contact with infected fomites and personnel, high animal density, high contact rates between domestic animals and wildlife, and lack of compliance with biosecurity measures [4–7], among others. Nevertheless, direct contact between infected and susceptible animals constitutes one of the principal mechanisms for FMD virus dissemination [6]. Importantly, this disease may spread among animals for long periods without showing any observable signs, imposing an additional challenge for devising prevention and control strategies [6].

Livestock movement is a common practice for most agricultural systems and links to trade and the need to access resources for animal sustainment [8, 9]. However, this practice increases the probability of direct contact between FMD-infected and susceptible animals despite its importance. Therefore, during the last years, several studies aimed to characterize how livestock movement features may serve as an indicator for the FMD dissemination risk [10].

The sanitary authorities at the country level must systematically gather information about livestock movements, inline with the recommendation of the World Organization for Animal Health in the International Animal Health Code [11]. More specifically, they must register information related to the official livestock trades [12, 13]. These records represent connections between livestock holdings, markets, and abattoirs, among others [14], which allow the construction of livestock transportation networks. The structure of these networks can critically influence the dynamics of transmission of many infectious diseases, including FMD [14].

In these transportation networks, the nodes represent places where animals may have contact with infectious agents, for instance, holdings or markets located in specific areas. The livestock movements between these correspond to edges [14, 15]. Different works characterized these transportation networks for previous FMD outbreaks, e.g., the well-documented 2001 United Kingdom FMD outbreak [16]. These studies identified that multiple nodal network properties related to disease spreading. For example, nodes with significant levels of FMD susceptibility reported high values for different centrality measurements, including in and out-degree, ingoing and outgoing infection chain, and betweenness, among others [14]. Other works on simulation also showed that some of these nodal centrality properties relate to infectious disease propagation [15, 17]. In principle, these local descriptions may allow the identification and prioritization of critical areas related to future FMD spread over time. However, despite its utility for describing some regional FMD epidemiological aspects, such a descriptive approach may be restricted to devising surveillance strategies because epidemiological vigilance requires the prioritization of the critical areas related to FMD [18]. This restriction emerges from the conflicting nature of the different centrality measurements [17], which likely result in different prioritizations of risk areas, limiting the possibility of constructing a unified rank for surveillance design. In addition, depending on the disease under study, network properties considered may differ. For instance, network properties related to fast spreading diseases, such as FMD, may vary from properties used to describe risk in bacterial origin diseases such as bovine brucellosis [19]. Therefore, computing a single high-risk area prioritization from multiple nodal movement network descriptions which reflect particular disease spreading properties constitutes a challenge not only for the disease characterization but also for devising cost-effective epidemic control alternatives [20, 21].

The main objective of this work was to construct an FMD risk regional transmission ranking based on livestock movement data and quantitatively evaluate how this ranking may benefit the prediction of future FMD disease spread. In contrast with previous works aimed to describe nodal network features likely related to virus transmission [17, 22], this work aims to use these nodal descriptions to construct a single ranking of FMD high-risk areas. The proposed ranking relies on super-spreaders, nodes that maximize their impact on other nodes during an outbreak [23], and which can be described by integrating multiple centrality nodal measures [24]. We hypothesized that a super-spreaders-based ranking (SSBR) may provide valuable information to address FMD in different stages, including early disease spreading and posterior disease proliferation [23]. To explore this hypothesis, we studied the prediction capacity of the SSBR of an actual FMD outbreak detected in the region of Cesar (Colombia). Our results show that the SSBR based on livestock movement data from 2016 helps prioritize areas subsequently affected by an FMD outbreak reported in 2018. The main contributions of our work are, first, the construction of a ranking of FMD high-risk areas based on cattle movement data and the notion of super-spreaders, and second, the quantitative evaluation of the prediction capacity of this ranking over an actual FMD outbreak. These findings may have implications for designing cost-effective planning mechanisms for disease breakout control and FMD epidemiology understanding.

## Materials and methods

**Fig 1:**
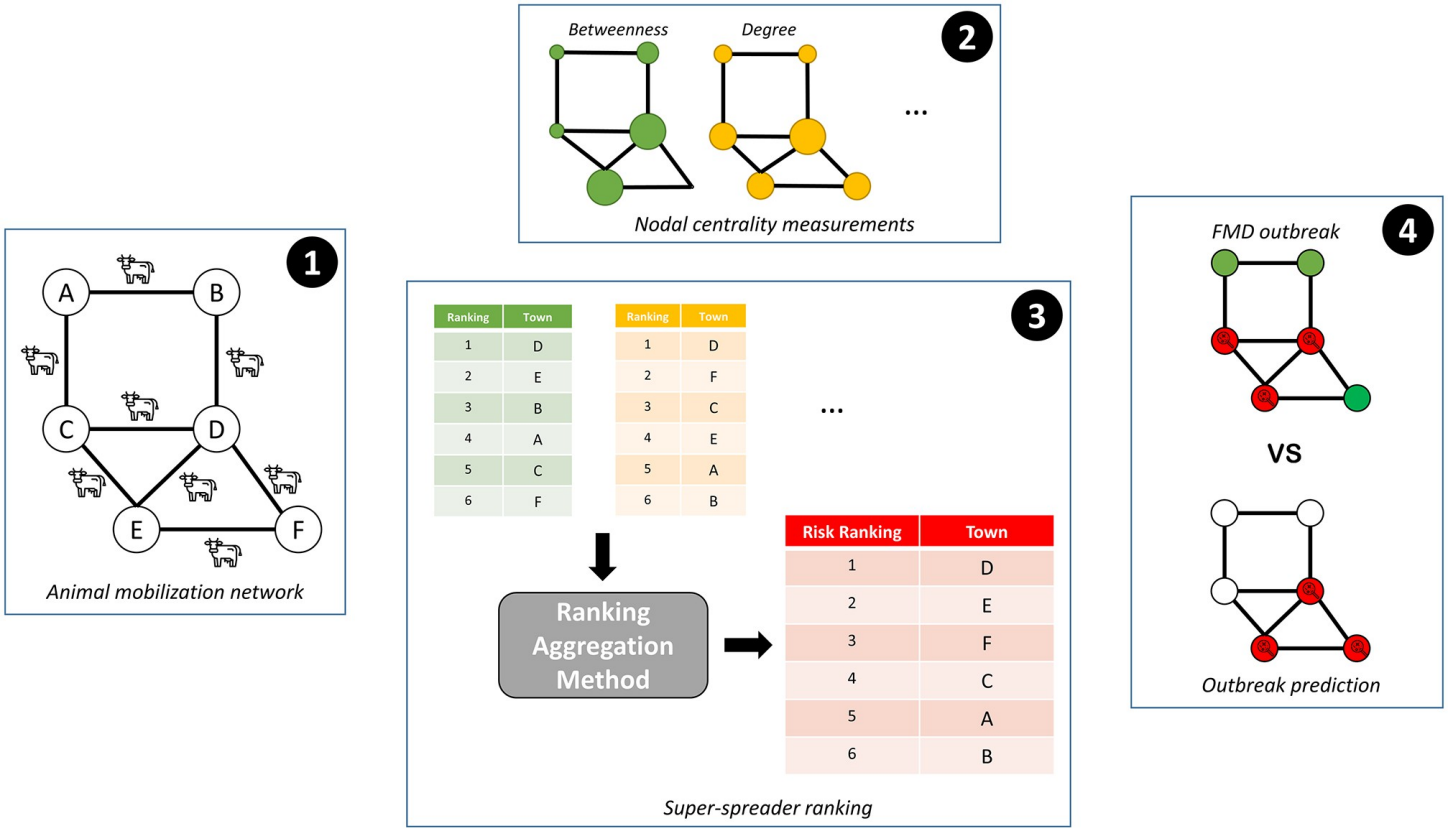
Workflow of the proposed methodology to compute rankings of FMD high-risk areas. First, cattle movement records allowed the construction of livestock mobilization networks. Then, centrality measurements, including betweenness and degree, allowed to build multiple rankings of risk. Next, a unique SSBR resulted from combining these rankings. Finally, based on the SSBR, predictions of possible future outbreaks were computed and compared against an actual FMD outbreak.

Fig 1 shows the methodology proposed for computing the ranking of areas with a high risk of FMD affectation. First, the mobilizations of animals on a daily scale were determined for the construction of cattle mobilization networks for non-overlapping monthly periods. Then, a set of nodal centrality properties were computed on these networks to describe different aspects of the FMD disease risk spreading. Next, these properties were combined to identify the super-spreader nodes and their corresponding SSBR. Finally, the predictive capacity of this ranking to detect an actual FMD outbreak was quantitatively evaluated.

### Livestock mobilization network

The information system for mobilization guides (SIGMA—Sistema de información para Guías de Movilización Animal—https://sigma.ica.gov.co/) provided livestock movement records used in this study. The Instituto Colombiano Agropecuario (ICA), the principal sanitary authority in Colombia administers this system. SIGMA contains the so-called *sanitarian guides of internal livestock mobilization*. These guides constitute the official authorization allowing cattle movements inside the country. Each guide includes the date, source, destination, species type, and the batch size of the livestock movements (number of transported animals). Due to use of organizational data, informed consent was not applicable.

This study focused on historical data of cattle movements from 1 January 2016 to 31 December 2016 in the Cesar (Colombia) region. This study concentrated on this region because later, in September 2018, it reported an FMD outbreak involving some of its municipalities [25, 26]. Therefore, this study aimed to predict these areas using the proposed ranking approach. The study conformed to guidelines of the Comite de Bioética at Universidad Nacional de Colombia, permitting exemption from full ethical review.

SIGMA mobilization data allowed the construction of livestock mobility networks. More specifically, the nodes corresponded to municipalities that served as the origin or destination of animal movements. A movement with at least one animal defined a link between nodes. The total number of animal mobilizations in non-overlapping monthly consecutive periods originating from the same municipality and ending at a different municipality characterized each link. This time partition size, i.e., about four weeks, assumes that it is unlikely that an FMD epidemic could persist without being identified beyond this time [14–16].

For the animal movement characterization, undirected, directed, and weighted directed networks of livestock movements were constructed [14]. Undirected networks described livestock movements without considering direction. Directed networks distinguished between the origin and destination of the livestock movement. Finally, weighted-directed networks accounted for the number of animals transported. Markets and holdings were considered different nodes in the graph. Data for different holdings were aggregated into a single holding per municipality. Livestock data movements for markets were also combined into a single marked node per municipality when they were present. Because of the low transmission risk in abattoirs, movements to these were not considered for the transportation graph construction.

### Super-spreading nodes identification

The underlying livestock movement network may serve as a proxy to study the animal contact phenomena. In particular, it may provide valuable information about the possible dynamics of the FMD disease spread [14]. The present analysis focused on identifying and ranking the so-called super-spreaders nodes [27]. These nodes maximize their epidemiological impact on other nodes [24]. Therefore, these nodes may link to a higher risk of FMD spreading and are worthy of being characterized.

#### Network centrality properties

Recent evidence suggests that the super-spreading phenomenon is closely associated with high centrality values in the nodes of the transportation network [24]. The work herein presented focused on particular nodal centrality measurements that capture livestock transportation features linked to these super-spreaders and that may increase FMD spreading chance.

Despite the large number of alternatives for nodal centrality measurements on these networks [28], only a few are good predictors of the super-spreader phenomena [17]. Therefore, this work concentrates on centrality described by degree and betweenness features, which proved to be good predictors of super-spreading [17]. This apriori selection of a few network features may also help minimize overfitting risk [29].

The degree of centrality corresponds to the number of connections each node has to other nodes [30]. In the livestock transportation network, the degree may describe the number of potential direct contacts per holding. For FMD, animals in nodes with a high degree, i.e., with many connections, are likely to become infected early in an epidemic outbreak [15]. The betweenness centrality estimates the extent to which a node lies on paths between other nodes by considering its importance for the shortest paths through the network [30]. In the FMD case, the nodes with high betweenness centrality are likely to accelerate the spread of infection through the network during livestock transportation [15]. Therefore, targeting nodes with higher degree and betweenness may help early identification and rapid disease control [24].

The risk of contagious and spreading FMD disease can be affected by the direction of the livestock movement. For instance, livestock movements toward abattoirs are less likely to spread diseases than livestock movements to markets [6]. Similarly, when the number of transported animals is large, the risk of contact with infected animals increases [6]. These two factors were accounted for by computing the degree and betweenness measurements in the three networks: undirected, directed, and weighted [31]. Depending on the kind of network, the definition of the centrality measurements may vary. For instance, in directed networks, two types of degrees may be considered, namely, in-degree and out-degree.

In summary, and accounting for the kind of network particularities, eight centrality measurements were computed for characterizing super-spreading behavior: 1) degree on undirected, 2) in-degree on directed, 3) out-degree on directed, 4) degree on weighted, 5) in-degree on weighted, 6) out-degree on weighted, 7) betweenness on undirected, and 8) betweenness on weighted. In principle, any of these measures computed on the corresponding transportation network, may provide a ranking of FMD risk for the nodes. However, previous work suggests that no single centrality measurements perform as the best predictor of disease spreading [24], mainly because different measures have different objectives. However, in most cases, epidemiological control requires a single rank for planning [24].

#### Super-spreaders identification

Borda’s count aggregation method provided a single super-spreader ranking for the nodes [24, 32]. This method employs as input a set of lists of ranks *R* = {*r*_1_, *r*_2_, ⋯, *r*_*n*_}. Each ranked list *r*_*i*_ has *K* items possibly in a different order, with *K* being the number of nodes in the transportation network. For the super-spreaders identification problem, the ranked lists resulted from computing the ascending order of the nodes for each centrality feature. Then the method defines a new rank based on an aggregated rank value defined for each element *j* as:

$$B\left(j\right)=\sum_{i\in R}K-r_{i}^{j}$$

where $r_{i}^{j}$ is the position of the *j*-th node in the rank *r*_*i*_. These *B*(*j*) are then reordered to provide Borda’s count rank.

As previously discussed, degree and betweenness characterization may have different epidemiological purposes, namely, modeling the potential first contagion and the possibility of disease propagation. In this work, these two objectives were modeled by using a hierarchical Borda’s aggregation. In particular, the degree (i.e., features 1) to 6) in Section) and betweenness-related ranks (i.e., features 7) and 8) in Section) were combined using Borda’s method, resulting in two different ranks. These two ranks were then combined again using Borda’s aggregation to construct the final aggregated SSBR.

### The 2018 FMD outbreak in Cesar (Colombia)

The super-spreaders’ approach may provide a single ranking suitable for prioritizing areas with higher FMD risk, more specifically, for quantifying FMD risk in areas where the disease is not present yet [33]. However, good risk indicators should help predict areas where this risk may materialize [33], i.e., the risk should be higher for regions later affected by FMD disease. Therefore, in contrast to previous studies that provided only risk descriptions, this study investigated the predictive capacity of the proposed ranking to predict regions for which FMD risk later materialized. For this, we used the mobilization data from 2016 to compute risk rankings and then studied its prediction capacity on an actual FMD outbreak detected in 2018 at the department of Cesar, Colombia.

**Fig 2:**
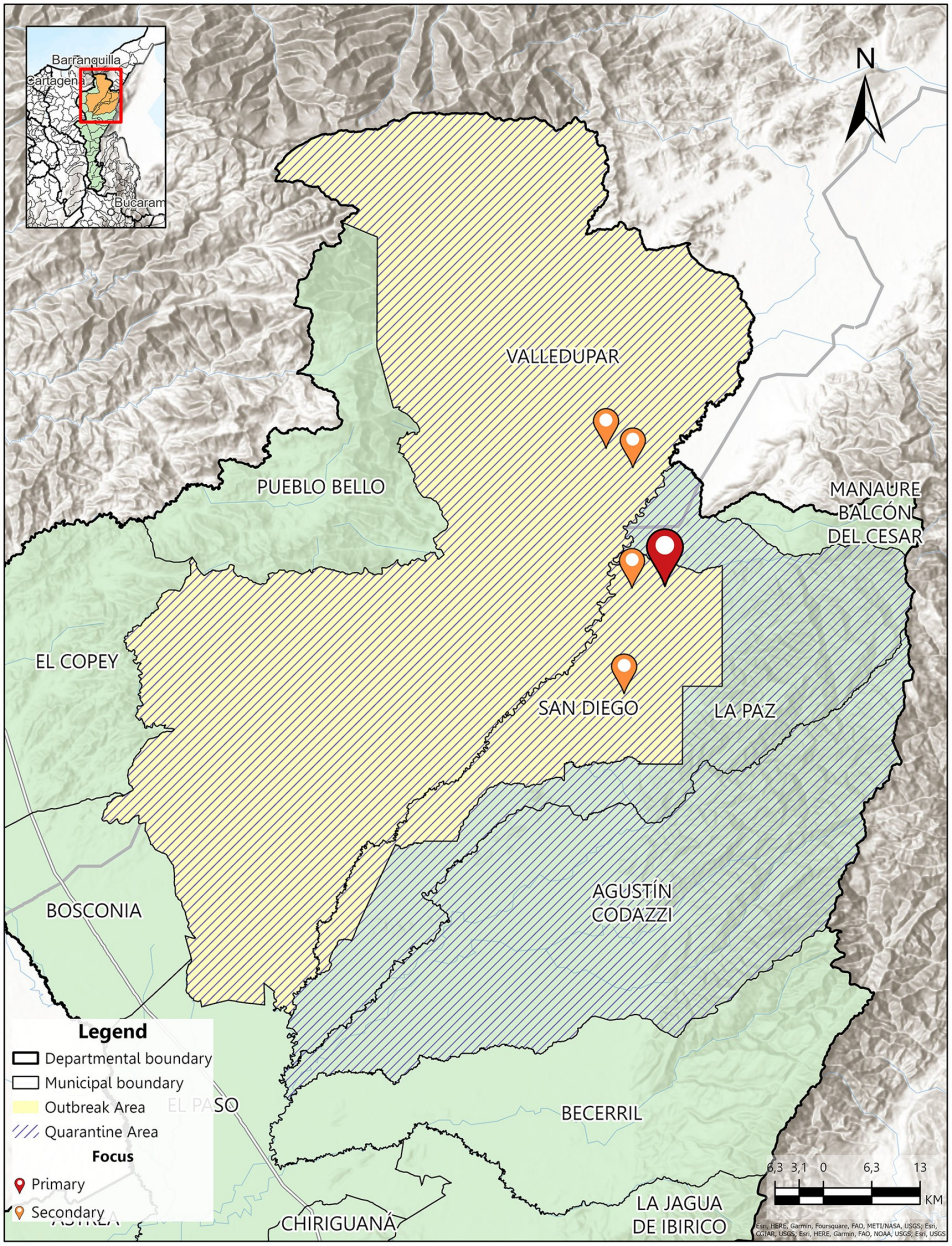
Municipalities affected by FMD in the Cesar (Colombia) department in 2018. Primary and secondary foci are marked as red and orange points, respectively. Quarantine (green) and outbreak (yellow) areas are also shown.

This outbreak was detected in October 2018 after two cows (from a holding of 216) tested positives for FMD type O in the municipality of San Diego [25]. Posteriorly, FMD infections were also detected in the municipality of Valledupar. The sanitary authority confirmed this infection, deployed epidemiological control protocols, and defined a quarantine control area [25, 26]. The area included the municipalities of Valledupar, La Paz (Robles), San Diego, and Agustín Codazzi, all located in Cesar (Colombia) [25, 26]. Officially, the outbreak originated with the illegal introduction of animals from the Bolivarian Republic of Venezuela. However, it cannot be discarded that there was a history of disease circulation in the area before the emergence of the outbreak [34]. Map at Fig 2 shows the municipalities affected by the 2018 outbreak at Cesar. In particular, primary (red) and secondary (organge) foci. We evaluated if the 2016 SSBR predicted the two FMD-affected municipalities in 2018.

Data were extracted and organized with Python 3.4.7. Calculations of measurements were performed using the Python module NetworkX 2.4. Super-spreader characterization, rankings, and municipality classification were computed using Python 3.4.7.

## Results

This work studied the construction of an network risk ranking based on the animal transportation network and its capacity to characterize areas affected by an actual FMD outbreak. First, we described the general features of the animal transportation data herein studied. Then, we reported the monthly SSBR computed using the 2016 transportation data. Following, we report these rankings’ predictive capacity in characterizing municipalities where the risk later materialized, i.e., the municipalities affected by FMD in 2018. We also studied the stability of the SSBR across different months. Finally, we studied the distributions for the centrality measurements considered.

### Livestock transportation network

A total of 21,254 livestock movements were reported between municipalities in the region of Cesar (Colombia) in 2016. Of these movements 9,854 (to markets and holdings) among 29 nodes in 25 municipalities were considered for the construction of the transportation network. A total of 175,068 animal movements were studied. Movements by truck were 97% and by walking, 3%.

### Network risk rankings

**Fig 3:**
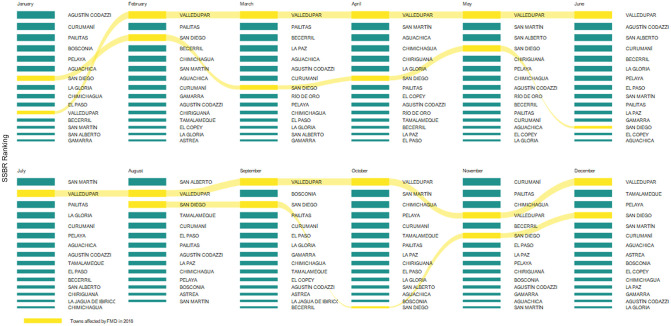
Network risk rankings computed for 2016 months. Each ranking corresponds to different municipalities displayed ordered depending on their level of risk. Yellow flow lines indicate the municipalities later affected by an outbreak in the same region in 2018.

Fig 3 shows the monthly SSBR (top 15) computed using the proposed approach using the 2016 livestock transportation data. The figure shows in yellow the municipalities later affected by the 2018 FMD outbreak. As observed, the proposed method ranks as high risk posteriorly affected by FMD or considered at high risk by the sanitary authority [25, 26]. More specifically, the proposed rankings tagged as high risk the municipalities posteriorly affected by the disease. S1 Table reports the municipalities ranked by the SSBR.

Valledupar, a municipality with an elevated risk in 2018, led the ranking most months [26]. San Diego, the municipality where the disease first emerged [25], showed also high the hierarchy throughout the year.

### SSBR predictive capacity to characterize materialized risk

Supervised classification allowed a quantitative evaluation of the rankings’ predictive capacity to characterize areas where the risk later materialized [35]. Specifically, we aimed to describe how well the SSBR obtained with 2016 livestock mobilization data predicted the municipalities that materialized the risk by FMD in 2018 [25, 26]. The orders induced by the 2016 centrality-based risk rankings provided the cut-offs for studying the true-positive rate or hit rate, i.e., the percentage of municipalities with materialized risk in 2018 correctly targeted by the 2016 proposed ranking from the total of municipalities in risk, and the false-positive rate or false alarm rate, i.e., the percentage of municipalities with materialized risk in 2018 incorrectly targeted by the 2016 rankings from the set of municipalities with no risk. The predictive ability of these classifiers was measured using the area under the receiver operative curve (ROC) [35]. For constructing this ROC curve, the number of municipalities prioritized by the proposed ranking was considered as the threshold. Additionally, to provide a quantitative measure of not highlighting a region affected by FMD. The negative predictive values (NPV) for monthly classifiers were computed. In particular, considering three classifiers constructed with the top-three areas provided by the SSBR, the mean and standard deviation of the NPVs across months were calculated. NPV was computed as True Negative/(False Negative + True Negative) [36].

**Fig 4:**
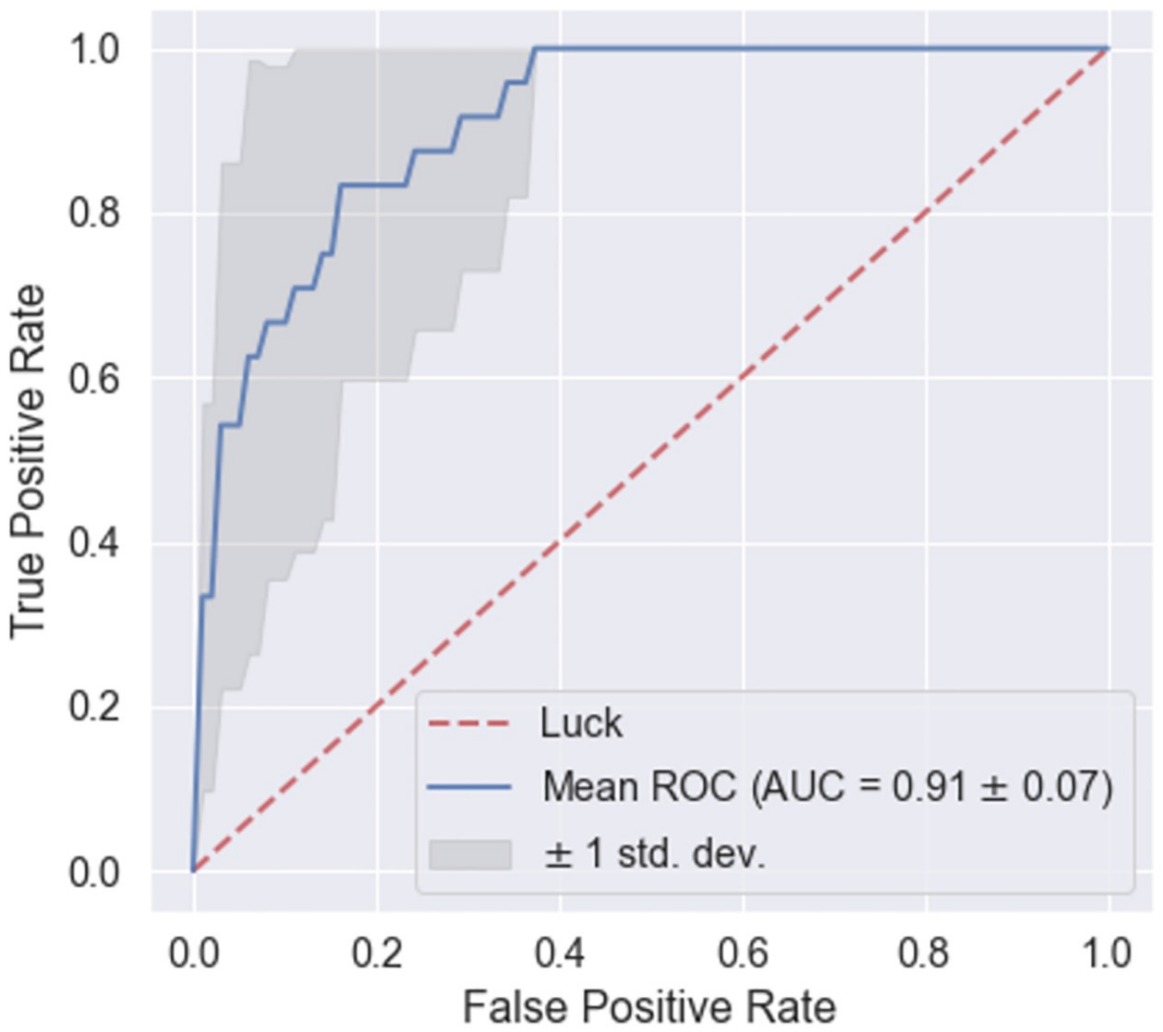
Mean and standard deviation of the ROC curves for different months. In this case, the task aimed to classify municipalities affected by the disease in 2018. Therefore, monthly ROC curves were constructed by considering varying cut-offs in the risk rankings proposed, i.e., changing the number of municipalities prioritized by the proposed ranking.

Fig 4 shows the mean of the ROCs for the months under study. As observed, the classifiers based on SSBR correctly targeted municipalities with high risk in 2018, with low false alarm rates. The mean of the area under the ROC for all the months was 0.91 ± 0.07 (*mean* ± 1*std*.*dev*.). The maximum area under the ROC was 0.98 in September, and the lowest performance resulted in July with 0.80.

The mean and standard deviation of the NPVs for the top-1 classifiers, i.e., risk classifiers that considered only the first region ranked by the SSBR were 0.66 ± 0.47. The mean and standard deviation of the NPVs for classifiers based on the top-2 ranking, i.e., risk classifiers relying on the first two regions ranked by the SSBR were 0.41 ± 0.18. At the same time, the mean and standard deviation of the NPVs for classifiers based on the top-3 ranking were 0.36 ± 0.21. Indicating that the proposed SSBR may miss some regions affected by FMD. For instance, see the month of July in Fig 3.

Finally, Table 1 reports the mean and standard deviation of the area under the ROC for the SSBR method and some commonly used alternative approaches for constructing risk rankings.

**Table 1:** The area under the ROC for five different methods for ranking regions likely affected by FMD. The proposed method SSBR combines different degree and betweenness features. The Borda degree and betweenness combine degree and betweenness features, respectively. Two rankings rely on the density of animals and the number of farms.

Ranking method	Mean	Standard deviation
*SSBR*	0.91	0.07
*Borda*′*sdegree*	0.9	0.06
*Borda*′*sbetweenness*	0.89	0.06
*Densityofanimals*	0.89	0
*Numberoffarms*	0.8	0

In particular, four different rankings were considered: two for degree and betweenness features, obtained by combining the corresponding features using Borda’s count aggregation, and two for the density of animals and the number of farms. As observed, the proposed approach improved disease characterization performance compared to other methods related to individual features and underlying productive variables.

### Rankings stability across months

Fig 3 shows that SSBRs varied across months, indicating that the risk may also change along the year, as livestock transportation dynamics may also vary during the year. To investigate these variations, we studied the stability of monthly rankings. For this, we computed Spearman’s rank-order correlation coefficient *ρ* between pairs of months [37], which assesses how well the relationship between two monthly FMD risks is described using the monotonic functions induced by the SSBR, i.e., the similarity of FMD risks obtained in the different months.

**Fig 5:**
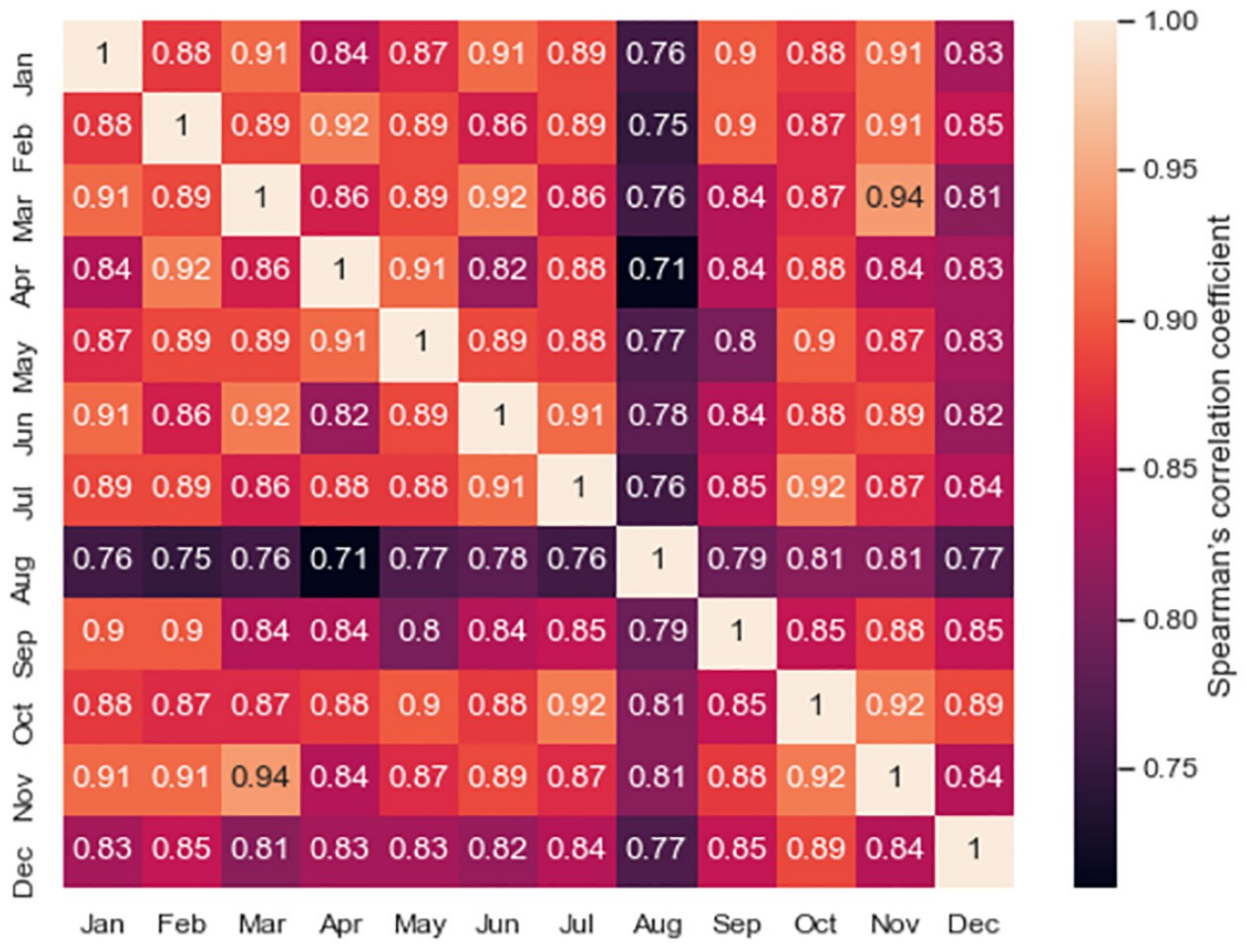
Spearman’s rank-order correlation coefficient of the FMD SSBR computed for pairs of months in 2016. High values on this measure represent high stable ranks.

Fig 5 shows the pairwise monthly stability measured by *ρ*. As observed, the FMD risk SSBR from January to July were highly stable, with stability values above 0.82. The stability obtained for August compared with the rest of the months decreased but remained high. The SSBR from September to December were highly stable, with values above 0.82.

### Distribution of centrality measurements

**Fig 6:**
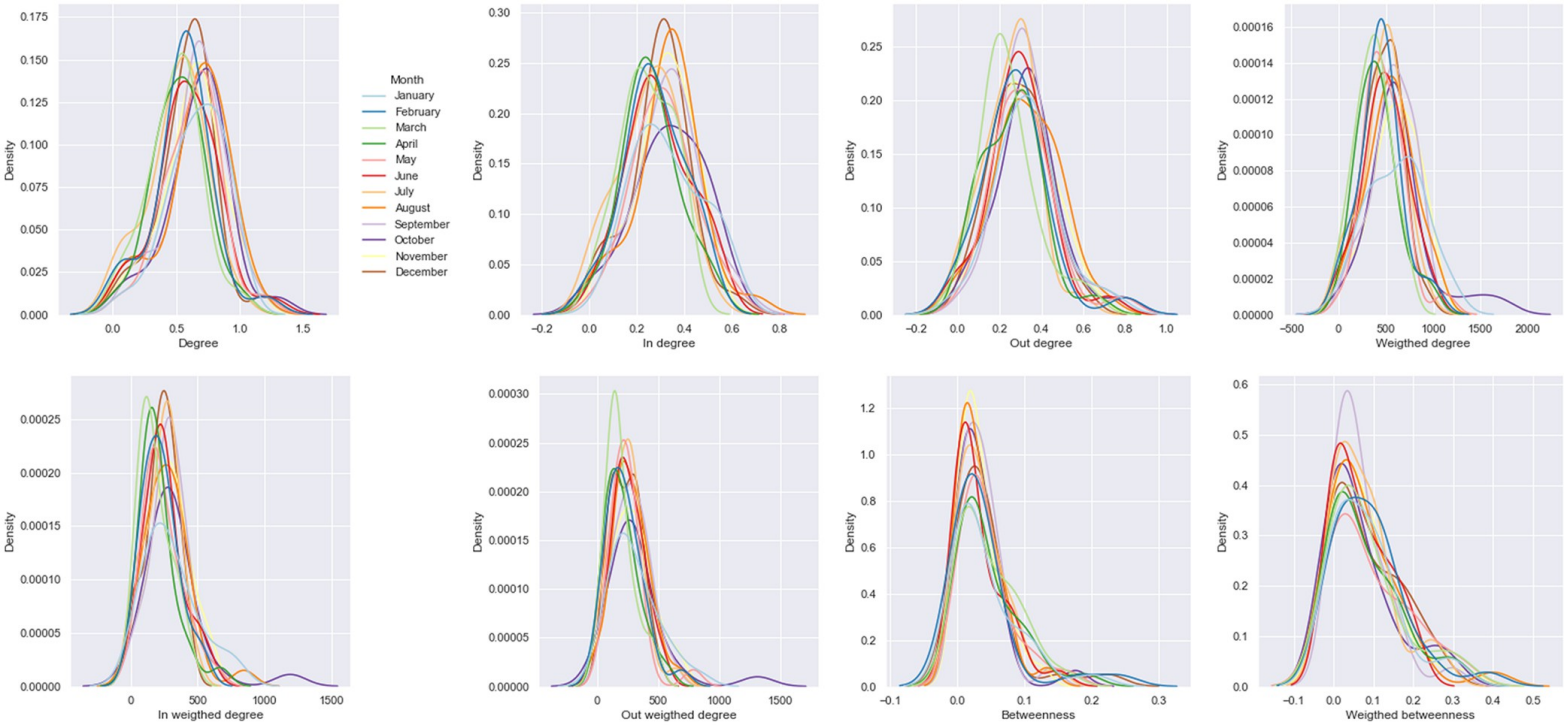
Distributions of different network centrality measurements. These distributions were computed per month for features related to degree and betweenness.

Fig 6 shows the distributions for the eight centrality measurements considered for the 2016 months. As expected, different centrality features exhibit different distribution shapes, reflecting specific livestock transportation network dynamic attributes.

For instance, degree distributions (Panel Degree at Fig 6) are more symmetric than betweenness distributions (Panel Betweenness at Fig 6), which have positive skewness and kurtosis values, likely related to the existence of nodes serving as intermediate in the network. Similarly, centrality measurement distributions change over months, evidencing a time-varying dynamic.

## Discussion

This work studied the construction of rankings of FMD risk from livestock transportation data and their predictive capacity to anticipate future disease outbreaks. A dataset consisting of more than 20,000 animal mobilizations from a region subsequently affected by FMD was analyzed using super-spreader rankings to describe the risk of contact. In contrast to previous livestock transportation network-based descriptions which mainly focused on features linked to FMD transmission, this study describes for the first time the construction of a single SSBR suitable for decision making, which can predict future outbreaks with high accuracy.

FMD is one of the most economically devastating livestock diseases because of the resulting production losses and the severe restrictions on the animal trade [2]. Therefore, developing strategies to anticipate and mitigate the disease is challenging for the productive sector and the sanitary authorities [38]. Ideally, these strategies should focus on high-risk areas in scenarios of limited resources [21, 39, 40]. Therefore, the prioritization of these areas is a significant requirement. Nevertheless, because of the complexity of the disease transmission mechanisms, the risks of FMD outbreaks are hard to establish [6]. Our results show that a single SSBR based on livestock transportation may provide high-performance values in determining future areas with FMD outbreaks. The simplicity of this ranking, essentially an ordered list of municipalities (see Fig 3), and its high predictive capacity for future outbreaks (see Fig 4) makes it suitable to be used in decision-making tasks.

Previous works on livestock transportation networks showed that high centrality values in nodes link to an increased risk of FMD transmission [14, 22]. However, although centrality is widely considered a valuable attribute for describing the risk, there is no consensus on how to define it [41]. Also, previous works show scenarios in which, depending on the disease nature (e.g. highly contagious) various centrality measures may result in different risk prioritizations [42, 43]. Therefore, using a single centrality to quantify the risk of nodes in the livestock transportation network threatens loss of insights obtained from other centrality measures. This work overcomes this limitation by combining multiple centrality measures, resulting in a single ranking, see Fig 1. Other authors proposed a similar approach for combining different nodal centrality measures methods, for instance, in the pig transportation network, to construct a risk index [40]. Our results complement these works by providing for the first time evidence of the predictive power of these indices in an actual outbreak. As Fig 4 shows, the use of the FMD risk index may successfully predict the areas with high risk in an actual outbreak. Importantly, our results suggest that this prediction capacity remains high for most months, implying that the livestock transportation-based index is quite informative about the materialization of FMD risk.

The proposed risk index may improve decisions regarding biosecurity and biocontainment in several ways. Some of the most common strategies to contain FMD outbreaks include culling, increasing biosecurity measurements, and vaccination, among others, all of which can benefit from this index [38]. For instance, in the case of FMD introduction, the proposed risk index may contribute to defining the culling area as part of the slaughter control policy [38]. In the case of an FMD outbreak, the need to act fast implies that culling should be performed on clinical and epidemiological grounds without the benefit of laboratory testing to confirm diagnoses [38]. Our results suggest that the FMD SSBR information may complement this evidence by being a good predictor of high FMD risk areas. Importantly, previous works indicate that removing these nodes reduces potential disease transmission [40]. Similarly, this FMD risk index may also inform prevention strategies, such as improved biosecurity and vaccination, by helping to focus and prioritize resources [21].

Fig 1 shows that the FMD risk index varied across months. However, the stability measure in Fig 5 suggests that the risk ranking is stable. Previous works have reported also changes in dynamic temporal networks [22, 40], but with a different approach. The common pattern is likely related to existing transport infrastructure and commercial network trades and the variations in productive and commercial dynamics [44]. For instance, the nodes identified in the analysis matched well with the road infrastructure, and some of them, including Valledupar, show the most intense cattle commercial activities. These variations can also be exploited, for instance, to guide the design of an epidemiological surveillance system focused on nodes with intense trade between them or to plan health programs by allowing selection of relevant nodes across time [45].

The proposed ranking corresponds to an ascending-ordered list of municipalities with risks related to FMD obtained per month (see Fig 3). Nevertheless, practical uses of these lists in epidemiology-related tasks, such as surveillance and disease control, require focusing resources on particular municipalities. Naturally, this selection may concentrate on the top areas of the ranking as they provide a higher predictive capacity of FMD risk, see Fig 4. However, it is worth recalling that the number of top municipalities highly depends on the particular application (for example, vigilance or control), type of resource, and resource capacities [46]. Therefore, the proposed ranking represents an additional input for improving planning in epidemiological tasks. Finally, strategies for determining the threshold (number of municipalities) from the ROC can be considered when there are no resource limitations [35].

This work has some limitations. First, it is worthy to remark that FMD risk indices are just one epidemiological tool that should be used in conjunction with other epidemiological information. Other relevant vulnerability factors, such as livestock production systems, market infrastructure, and the biophysical environment, should inform FMD control policies [47]. Second, illegal trades may increase transmission risk, especially within international borders [48]. Our work is based only on official exchanges registered by the sanitary authority, providing a limited view of the animal transportation risk. In addition, our analysis also ignores the existence of alternative transmission pathways, for instance, induced by the movement of people or fomites [49]. Future work should explore the characterization of the risk linked to these illegal trades and these alternative transmission pathways. Finally, the unit of analysis, i.e., the municipality, is quite large. Future work may include a detailed analysis of movements among farms, abattoirs and markets.

## Conclusion

We studied the construction of network risk rankings to describe livestock mobility. For this, we proposed the construction of a monthly livestock mobility network characterized by different centrality measurements that capture features related to disease transmission. These measurements were combined into a single ranking to describe super-spreaders on the transportation network. Our results show for the first time that the rank computed on historical data provides a high predictive capacity for future FMD outbreaks. The proposed rankings vary across months, likely linking to variations in the commercial animal dynamic. However, the stability of rankings across most of the year is high. Further work should integrate these ranks with other epidemiological information sources to devise cost-effective control policies for FMD.

## Supporting information

S1 Table Rankings of municipalities computed for 2016 months. (XLS)
