# Correction: A foot and mouth disease ranking of risk using cattle transportation

**DOI:** 10.1371/journal.pone.0294615

**Published:** 2023-11-14

**Authors:** Fausto Moreno, Juan Galvis, Francisco Gómez

In subsection Super-spreading nodes identification from section Materials and Methods, a reference is omitted from the second sentence of the second paragraph.

The correct sentence is: The work herein presented focused on particular nodal centrality measurements that capture livestock transportation features linked to these super-spreaders and that may increase FMD spreading chance [1].

The reference is: Gómez, Francisco, et al. “Identification of Super-Spreaders of Foot-and-Mouth Disease in the cattle transportation network: The 2018 outbreak case in Cesar (Colombia).”; 2019 4th World Conference on Complex Systems (WCCS). IEEE, 2019.
